# Functional outcome measures in a surgical model of hip osteoarthritis in dogs

**DOI:** 10.1186/s40634-016-0053-5

**Published:** 2016-08-15

**Authors:** Dianne Little, Stephen Johnson, Jonathan Hash, Steven A. Olson, Bradley T. Estes, Franklin T. Moutos, B. Duncan X. Lascelles, Farshid Guilak

**Affiliations:** 1 Department of Orthopaedic Surgery, Duke University Medical Center, 375 MSRB 1, BOX 3093 DUMC, Durham, NC 27710 USA; 2 Comparative Pain Research Laboratory and Comparative Medicine Institute, Department of Clinical Sciences, North Carolina State University College of Veterinary Medicine, Raleigh, NC USA; 3 Cytex Therapeutics Inc, Durham, NC 27705 USA; 4 Department of Orthopaedic Surgery, Washington University and Shriners Hospitals for Children - St. Louis, St. Louis, MO 63110 USA; 5 Department of Basic Medical Sciences, Purdue University College of Veterinary Medicine, 625 Harrison St West Lafayette, IN, USA

**Keywords:** Synovium, Inflammation, Collagen, Proteoglycan, Pain, Tissue engineering, Articular cartilage repair

## Abstract

**Background:**

The hip is one of the most common sites of osteoarthritis in the body, second only to the knee in prevalence. However, current animal models of hip osteoarthritis have not been assessed using many of the functional outcome measures used in orthopaedics, a characteristic that could increase their utility in the evaluation of therapeutic interventions. The canine hip shares similarities with the human hip, and functional outcome measures are well documented in veterinary medicine, providing a baseline for pre-clinical evaluation of therapeutic strategies for the treatment of hip osteoarthritis. The purpose of this study was to evaluate a surgical model of hip osteoarthritis in a large laboratory animal model and to evaluate functional and end-point outcome measures.

**Methods:**

Seven dogs were subjected to partial surgical debridement of cartilage from one femoral head. Pre- and postoperative pain and functional scores, gait analysis, radiographs, accelerometry, goniometry and limb circumference were evaluated through a 20-week recovery period, followed by histological evaluation of cartilage and synovium.

**Results:**

Animals developed histological and radiographic evidence of osteoarthritis, which was correlated with measurable functional impairment. For example, Mankin scores in operated limbs were positively correlated to radiographic scores but negatively correlated to range of motion, limb circumference and 20-week peak vertical force.

**Conclusions:**

This study demonstrates that multiple relevant functional outcome measures can be used successfully in a large laboratory animal model of hip osteoarthritis. These measures could be used to evaluate relative efficacy of therapeutic interventions relevant to human clinical care.

**Electronic supplementary material:**

The online version of this article (doi:10.1186/s40634-016-0053-5) contains supplementary material, which is available to authorized users.

## Background

The hip is one of the most prevalent sites in the body for osteoarthritis, and over 330,000 total hip arthroplasties are performed in the USA each year. Over half of these surgeries are in individuals less than 64 years old (CDC 2010), and the incidence of hip arthroplasty in patients under the age of 50 is increasing (Skytta et al. 2011). A major clinical concern is that revision rates of total hip arthroplasty (THA) are nearly 50 % in this age group (Baker et al. 2011) (Kim et al. 2011), and revised implants have low survivorship and high complication rate compared to primary hip arthroplasty (Adelani et al. 2013). This has led to substantial interest in the development of new therapeutic strategies to improve on results of hip arthroplasty or to delay the need for these procedures (Guilak 2010; Lee et al. 2010; Warnke 2010; Field et al. 2011; Hauser and Orlofsky 2013; Schauss et al. 2012; Vilar et al. 2013).

Of particular interest is the growing potential for the use of stem-cell-based therapies for osteoarthritis, such as total joint resurfacing using a tissue-engineering approach (Diekman and Guilak 2013; Lee et al. 2010; Hung et al. 2003). The requirements for a pre-clinical model of osteoarthritis in which these approaches could be tested are rigorous, since the gold standard and current standard of care is the highly successful hip arthroplasty procedure. Therefore, the burden of proof for beneficial clinical and functional outcomes of any new hip osteoarthritis treatment is high, and high predictive validity of any pre-clinical model is essential (Henze and Urban 2010; Little and Hunter 2013; Piel et al. 2013). Ideally, such a pre-clinical model should allow comparison of functional outcome data back to a natural disease state or a well-established treatment such as THA.

A number of canine models and outcome measures have been used to evaluate implants for THA (Mendes et al. 1974; Skinner and Mabey 1987; Skurla and James 2005; Skurla et al. 2005); for example, gait analysis outcomes have been correlated with the histological scores of the joint capsule (Mendes et al. 1974; Skinner and Mabey 1987). Dogs also commonly develop naturally occurring hip osteoarthritis, which is treated by THA and evaluated by clinical parameters and functional outcome measures comparable to those used to evaluate results after THA in humans (Lascelles et al. 2006, 2010b; Dow et al. 2009; Hansen et al. 2007; Risler et al. 2009; Morton et al. 2005; Brown et al. 2008). Thus a large population of dogs with naturally occurring hip osteoarthritis treated by THA exists for comparison of functional outcomes after any new therapeutic intervention. In post mortem retrieval studies following THA to treat clinical disease in dogs (Skurla and James 2005; Skurla et al. 2005), the mechanism of implant wear was remarkably similar to that seen in humans. Early failure events were deemed to be mechanical rather than biological in nature, and were suggested to originate from high activity levels in dogs postoperatively. Together, these data suggest that a canine model could be helpful in assessing tissue engineering approaches that are particularly relevant to younger and more active human patients.

In addition to pre-clinical studies to evaluate THA components in normal joints, attempts have been made to induce osteoarthritis in canine models by performing pelvic osteotomies to alter the acetabulum-femoral relationship (Inerot et al. 1991; Renberg et al. 2000). In these models, the joint capsule is not entered but joint mechanics and loading mimic hip dysplasia, with reduced acetabular coverage of the femoral head, and development of osteoarthritis may be inconsistent. Alternatively, debridement of cartilage from the femoral head and acetabulum in sheep induces moderately severe hip osteoarthritis (Phillips and Gurr 1989) and resulted in few complications, even though in these studies the use of capsulorrhaphy was not reported (Phillips et al. 1990). However, hip arthroplasty surgery involves arthrotomy, and joint capsule stability is critical for prevention of post-arthroplasty dislocation (Chivas et al. 2006; Tsai et al. 2008; White et al. 2001; Hayes et al. 2011) and for return to a consistent gait (Holnapy et al. 2013). These data led us to evaluate femoral head cartilage debridement with the use of capsulorrhaphy to maintain joint stability and prevent post-operative dislocation or chronic subluxation as a method of inducing osteoarthritis in the dog hip. In future studies, maintenance of joint stability post-operatively could also allow the effect of novel therapeutics to be better evaluated, without masking of treatment effects by post-operative instability. The objective of this study was to develop a model of hip osteoarthritis in the dog that would induce functional disability and radiographic abnormalities similar to those identified in humans and canines with naturally occurring osteoarthritis presenting for THA. Outcome measures relating to function (accelerometry, gait analysis), structure (radiography, goniometry, limb circumference, histology) and pain (pain and function scoring, gait analysis) were evaluated. Of these outcome measures, radiography, dynamic gait analysis and histology represent those most commonly used previously in research studies, whilst in naturally occurring osteoarthritis in the dog, accelerometry, static and dynamic gait analysis, radiography, goniometry, limb circumference and functional scoring all form part of a comprehensive clinical examination and were evaluated to provide a baseline for future studies in this osteoarthritis model.

## Methods

### Animals

All animal procedures were approved by the Duke University Institutional Animal Care and Use Committee. Purpose-bred intact male hounds (*n* = 7, 26–32 kg at the time of surgery) were purchased at 4–6 months of age from an approved vendor and were socialized, trained to leash exercise and acclimated to cages with height modification. Radiographic evidence of epiphyseal growth plate closure in the proximal femur was obtained at 10–12 months of age. Animals underwent clinical examination, radiography and outcome measure testing (described herein) 2 weeks preoperatively to obtain baseline data and to screen for preexisting developmental hip dysplasia. For any dog that was determined to have preexisting dysplasia, the least affected hip was selected for surgical intervention, since preexisting soft tissue laxity resulting from hip dysplasia is a known risk factor for dislocation after canine THA (Hayes et al. 2011).

### Surgical procedure

Animals were administered peri-operative analgesia (pre- and intra-operative transdermal fentanyl (75–100 μg/h), postoperative transition to oral tramadol (2–5 mg/kg PO q8hours) for 21 days postoperatively), antibiotics (intra-operative cefazolin (22 mg/kg IV), and epidural (bupivacaine <0.5 mg/kg /morphine <0.1 mg/kg in total volume not to exceed 0.1 mL/kg)) and general anesthesia (propofol (2–8 mg/kg IV to effect)/isoflurane (0.5–4 % administered in O_2_ via endotracheal tube with intermittent positive pressure ventilation). A standard craniolateral approach to the craniodorsal aspect of the selected hip joint was performed (Piermattei and Johnson 2004). The ligament of the head of the femur was disrupted, and the hip joint was dislocated. Remnants of ligament and cartilage were debrided from the cranio-dorsal aspect of the femoral head using a Dyonics PowerMax Shaver and Dyonics 4.5 mm Slap Burr (Smith & Nephew, Andover, MA). Debrided cartilage debris was removed using continuous lavage and suction. A total of approximately 25–30 % of the surface area of the femoral head was debrided of cartilage to the level of subchondral bone using the Slap Burr, and both the femoral and acetabular remnants of the ligament of the head of the femur were sharply debrided flush with the articular surface using a scalpel. Capsulorrhaphy was performed and reinforced with two 2.7-mm titanium bone anchors (Securos, Fiskdale, MA), with two sutures of 2 Tigerwire (Arthrex, Naples, FL) passed through a 1.5-mm transosseous tunnel, as described previously (Piermattei et al. 2006). Animals were maintained on postoperative analgesia and strict cage rest for 3 weeks postoperatively. Beginning at 4 weeks postoperatively, dogs were permitted 10 min/day of leash exercise but were otherwise maintained in height-limited kennels to prevent jumping, and no unrestrained exercise was permitted for the entire postoperative period. The contralateral hip was maintained as an un-operated control.

### Radiography, goniometry and limb circumference

Multiple radiographic views were obtained on each animal preoperatively and at 1, 4, 8, 12, 16, and 20 weeks postoperatively under dexmedetomidine (500 μg/m^2^ IM) and butorphanol (0.2 mg/kg IM) sedation. Radiographs were evaluated for compression indices (CIs) and distraction indices (DIs) of each hip as measures of joint congruency and joint laxity, respectively (Runge et al. 2010; Gold et al. 2009; Todhunter et al. 2003). Extended ventro-dorsal projections were used to calculate preoperative Norberg Angle and estimate femoral head coverage using the scoring system developed by the Orthopedic Foundation for Animals (OFA) (www.ofa.org) (Gold et al. 2009; Hou et al. 2010). These preoperative radiographic indices were used to select the most normal hip for surgery. CIs and DIs were monitored in both hips throughout the postoperative period as a measure of change in joint congruency and joint laxity in response to surgery. The extended ventro-dorsal projection was used to grade each hip joint for osteoarthritis, using a modification of the grading system proposed by Lane (Lane et al. 2004). Osteophyte scores in each of four joint locations (craniolateral and caudomedial femoral head and cranial and caudal acetabulum) were assigned on a scale of 0–3 in each location (0 = no osteophyte, 1 = possible osteophyte, 2 = definite osteophyte, 3 = severe osteophyte). Subchondral cysts, sclerosis and femoral head deformity were scored individually (0 for absent and 1 for present) as described previously (Lane et al. 2004), and additional scores (scored 0 for absent and 1 for present) were assigned for circumferential linear femoral head osteophytes and caudal curvilinear femoral neck osteophytes as has been used in the early radiographic detection of canine hip dysplasia (Risler et al. 2009). Thus a summed joint radiographic osteoarthritis score (range 0–17) was assigned for each joint (operated vs. control) at each time point as well as pre-operatively. All radiographs were graded blindly by two experienced veterinarians (DL and BDXL). During sedation for radiography, goniometry was used to assess maximal flexion and extension of both operated and control hip joints, and the circumference of each thigh was measured at the level of the plica lateralis. For these procedures, measurements were obtained in triplicate for each limb and the mean value was used for analysis. The same investigator performed all measurements.

### Pain and function scoring

The Glasgow Composite Measure Pain Scale – Short Form (CMPS-SF) was used to score pain pre- and peri-operatively every 6–8 h, then weekly for the duration of the study (Morton et al. 2005; Valtolina et al. 2009; Holton et al. 2001). The Canine Brief Pain Inventory (CBPI), a scale validated for the assessment of osteoarthritis-associated pain and activity impairment in clinical cases when completed by owners familiar with the dog was completed for each dog preoperatively and at 2, 4, 8, 12, 16, and 20 weeks postoperatively. The mean score of three independent graders was calculated and reported for each dog at each time point (Brown et al. 2008, 2009).

### Accelerometry

Accelerometry-based monitors (Actical®, Philips Respironics, Bend, OR) were used to record activity for 7 consecutive days preoperatively, and 1, 2, 4, 6, 8, 10, 12, 16, and 20 weeks postoperatively. Data were analyzed by custom code in Matlab (Mathworks, Natick, MA), including mean total day time (06:00–18:00) and night time (18:00–06:00) activity, number and duration of transitions from activity to rest period during the day time and night time periods, and the mean activity per minute over 24-h to evaluate circadian activity patterns.

### Gait analysis

A pressure sensitive walkway (PSW) (7100 QL Virtual Sensor 4, Tekscan, Boston, MA) was used for gait analysis. This system has been validated previously for use in normal dogs, dogs with osteoarthritis, and dogs before and after THA (Lascelles et al. 2006, 2010b; Wernham et al. 2011). Before each use, the PSW was calibrated at start-up in both dynamic and static modes. Dynamic and static datasets were acquired preoperatively and every 4-weeks postoperatively until sacrifice. Dynamic kinetic data were collected as described previously (Lascelles et al. 2006; Seibert et al. 2012). Ten valid runs were collected for each dog at each time point, and velocity (measured directly from PSW data) was restricted to a range of 1.77–2.65 m/s, and acceleration to 0.3 m/s^2^. Unfortunately, different PSWs had to be used for collection of data between dogs and across time points, therefore to normalize for the differences between the PSWs, dynamic data were evaluated by calculation of between-limb ratios of peak vertical force, vertical impulse and maximum peak pressure and duty factor (the fraction of the duration of the stride for which each foot remains on the ground). Static body weight distribution data were collected as previously described (Lascelles et al. 2006, 2010b; Wernham et al. 2011), and these data points could be used as collected since between limb comparisons conducted at the same time were unaffected by the use of different PSWs. Ten valid sets of data were collected for each dog at each time point. For each data set, the mean percentage bodyweight distribution through each limb over the most steady 5-s period was recorded.

### Histological evaluation

Dogs were euthanized 21 weeks after surgery with an overdose of pentobarbital sodium and phenytoin sodium (Beuthanasia D solution, Schering-Plough Animal Health Corp., Union, NJ). Femora and pelves were harvested intact, wrapped in phosphate-buffered saline (PBS)-soaked gauze and frozen (−20 °C) until analysis. Synovium was harvested from the cranial, caudal, medial and lateral joint capsule, fixed in 10 % formalin, sectioned and stained with Hematoxylin and Eosin. Stained sections were evaluated by four blinded graders and scored according to a modification of a canine knee synovial grading system (Cook et al. 2010) (Additional file 1: Table S1). Acetabulum and femoral head of each joint were sectioned into two halves using a band saw, then fixed for 48 h in 10 % formalin and placed in Decal Overnight solution (Fisher Scientific) for 4–5 days until each half could be cut into 4 additional circumferential pieces using a scalpel to yield eight circumferential blocks for the acetabulum and femoral head. Decalcification of blocks was completed in 10 % EDTA solution (pH 7.4) at 37 °C for 10–14 days, and they were then were dehydrated and paraffin embedded. Eight micron sections were cut and stained with Safranin-O/fast green. Osteochondral sections were scored using a modified semi-quantitative scoring system (Additional file 1: Table S1) (Elliott et al. 2002).

### Data analysis

All data were reported as mean ± SD. All statistical analyses were performed using statistical software (Statistica, StatSoft). For statistical analysis, data were evaluated for normality, equal variance and sphericity, and analyzed by one-way repeated-measures analysis of variance with multiple dependent measures (MANOVA). Tukey’s post-hoc test was used to determine differences between measures following MANOVA if the main effect was significant. For data that failed these assumptions, the non-parametric Kruskal-Wallis test was used to compare differences between preoperative and postoperative values within each limb, and Wilcoxon’s matched pairs test was used to identify differences between control and operated limb at each time point. Spearman rank order correlations were used to evaluate relationships between histological scores and clinical and functional outcome measures. Significance was reported at the 95 % confidence level (α = 0.05).

## Results

All dogs completed the study without major complications or need for additional analgesia. Incisional interference resulted in subcutaneous seroma formation in one dog and partial skin incision dehiscence in a second dog, both of which were managed conservatively.

### Radiography

**Fig. 1:**
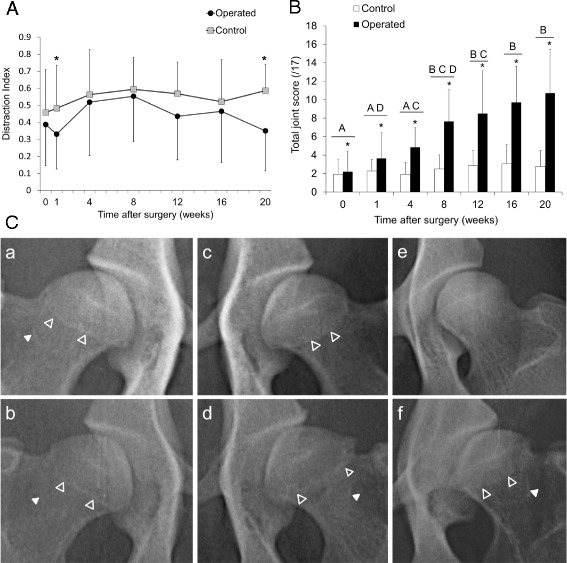
(**A**) Distraction index of operated and control limbs as assessed by radiography. *Operated significantly less than control limb (*p* = 0.04). (**B**) Modified Lane radiographic scores of operated and control limbs. *Significantly greater score than control limb (*p* = 0.022). *Bars* sharing the same *letters* are not significantly different from each other (*p* < 0.05), *n* = 7. (**C**) Preoperative (*a*,*c*) and 20-week (*b*,*d*) radiographs of control (*a*,*b*) and operated (*c*,*d*) limbs. Range in radiographic appearance of preoperative femoral head phenotype from normal (*e*) to dysplastic (*f*). *Open arrow heads* indicate circumferential linear femoral head osteophytes, and *closed arrow heads* indicate caudal curvilinear femoral neck osteophytes

According to the Orthopedic Foundation for Animals scoring system, only 5/14 hips (3/7 dogs) had good hip conformation preoperatively. Norberg angle was >105 ^o^ in 2/14 hips (1/7 dogs), and DI was ≤0.3 in 4/14 hips (2/7 dogs). Thus the majority of animals used in the study had preexisting bilateral hip dysplasia. There was no significant difference in DI (laxity) postoperatively compared to preoperative values for control and operated limbs, but operated limbs had a significantly lower DI (less laxity) than control limbs at 1 and 20 weeks postoperatively (*p* = 0.04) (Fig. 1A). CI (congruency) was not significantly different between control and operated limbs or between any pre- and postoperative time points. Radiographic osteoarthritis score was significantly greater in operated than control limbs, demonstrated a progressive increase over time and was significantly increased from preoperative values beginning at 8 weeks postoperatively (Fig. 1B and C). However, substantial variation in control limb phenotype was observed radiographically due to preexisting hip dysplasia (Fig. 1C).

### Goniometry and limb circumference

**Fig. 2:**
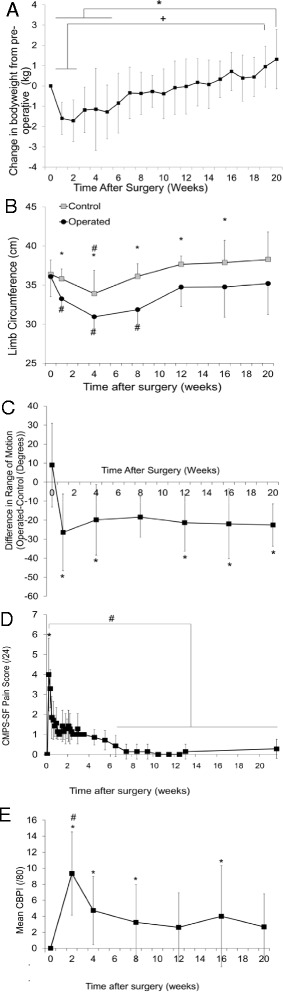
**a** Change in bodyweight compared to preoperative values. *Week 20 differs from weeks 1–5 (*p* = 0.03). ^+^Week 19 differs from weeks 1 and 2 (*p* = 0.03). **b** Pre- and postoperative limb circumference in control and operated limbs. *Control and operated are different (*p* < 0.007). ^#^Significantly different from preoperative within the same limb (*p* < 0.03). **c** Difference in total range of motion between control and operated limbs pre- and postoperatively. *Different from time 0 (*p* < 0.04). **d** Glasgow Composite Measure Pain Scale – Short Form. *Day 1 different from day 0 (*p* < 0.0001). ^#^Weeks 6–21 different from day 1 (*p* < 0.02). **e** Canine Brief Pain Inventory. ^#^Different from all other time points (*p* < 0.001). *Different from preoperative (*p* < 0.04)

Bodyweight (Fig. 2a) did not significantly change from preoperative values through the duration of the study but was significantly lower in the first 5 weeks postoperatively than at 20 weeks (*p* = 0.03). Limb circumference (Fig. 2b) was significantly decreased in operated compared to control limbs from the first postoperative week until week 16, and there was a trend (*p* = 0.07) for continued disparity at week 20. Compared to preoperative values, operated limbs demonstrated a decrease in circumference for the first 8 weeks postoperatively, whereas this was only evident in control limbs at 4 weeks postoperatively. Range of motion decreased in the operated limb compared to control for every postoperative time point except at 8 weeks (Fig. 2c).

### Pain and function scoring

Scores obtained using the CMPS-SF (Fig. 2d) were increased in the immediate postoperative period, but returned to baseline values and were not different from preoperative values after week 6. Scores obtained using the CBPI (Fig. 2e) demonstrated immediate postoperative elevation and remained elevated for the majority of the study, indicating continued assessment of functional impairment by caregivers compared to preoperative values.

### Accelerometry

**Fig. 3:**
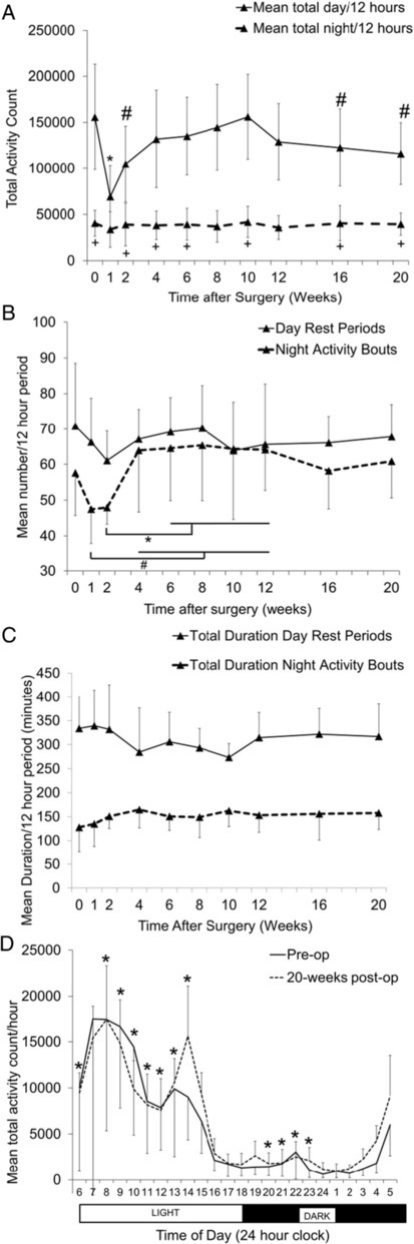
**a** Mean total daytime (*light phase*) and nighttime (*dark phase*) activity counts for each 12-h time period over a 7-day recording period. *Significantly different from every other day data point (*p* < 0.02). ^#^Different from time 0 day (*p* < 0.04). ^+^Same as week 1 day (*p* > 0.05). **b** Mean number of daytime (*light phase*) rest periods and nighttime (dark phase) activity bouts for each 12-h cycle over a 7-day recording period. *p < 0.04, ^#^ < 0.05. **c** Mean total duration of daytime (light phase) rest periods and nighttime (*dark phase*) activity bouts over each 12-h cycle over a 7-day recording period. **d** Mean total activity count each hour over a 24-h cycle and 7-day recording period—one-way within-subjects analysis of variance with multiple dependent measures (time of day and postoperative time). *Different between preoperative and 20 weeks postoperatively within subject

Accelerometry data demonstrated significant reduction in total day-time activity counts in the first 2 postoperative weeks and were not different from total night-time activity counts in the first postoperative week (Fig. 3a). By 4 weeks postoperatively, day-time activity counts returned to baseline but decreased again at 16 weeks until the end of the study. Total night-time activity counts were not affected by surgery. In contrast, number of day-time rest periods in each 12 h period did not change significantly post-operatively, but the number of bouts of activity during the nighttime sleep period was increased when comparing 1 week postoperatively with 4–12 weeks postoperatively, and 2 weeks postoperatively compared to 6–12 weeks postoperatively (Fig. 3b). However, total duration of daytime rest periods or nighttime bouts of activity in each 12-h period did not change significantly over time (Fig. 3c). On closer inspection of the data, the group of seven dogs appeared to cluster into two different behavioral subgroups. Three dogs were more active preoperatively, rested more during the day postoperatively and had reduced overall postoperative activity compared to preoperative values. The four remaining dogs were less active preoperatively, rested less during the day postoperatively and had increased overall postoperative activity compared to preoperative values (Additional file 2: Figure S1A). However, the study was underpowered to detect significant differences between these behaviors within the overall group (Additional file 2: Figure S1B). Data were evaluated by one-way within-subjects analysis of variance with multiple dependent measures (time of day and postoperative time) for differences over a 24-h light/dark cycle preoperatively and at 20 weeks postoperatively (Fig. 3d). There were significant disruptions in activity level between 8 am and 2 pm and from 8 pm to 11 pm between preoperative and 20 weeks postoperative time points.

### Gait analysis

**Fig. 4:**
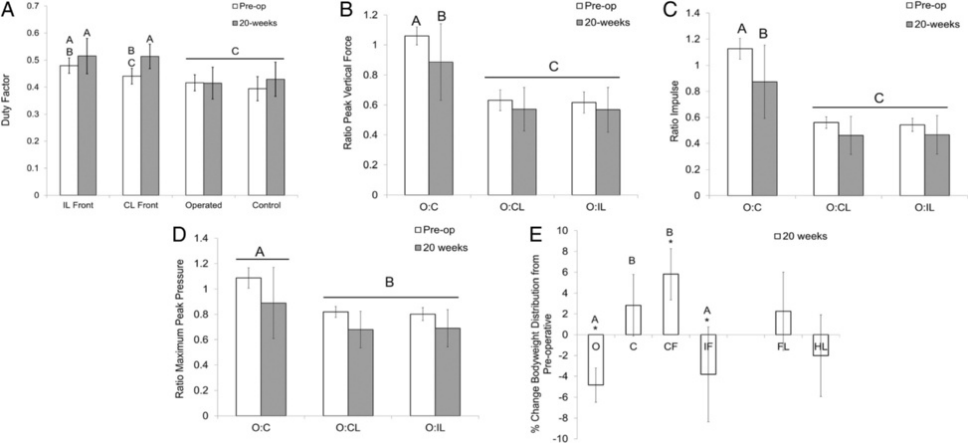
**a** Duty factor for each limb preoperatively and at 20 weeks postoperatively. IL = ipsilateral forelimb (same side forelimb); CL = contralateral forelimb (diagonal forelimb). **b** Ratio of peak vertical force for operated limb compared to all other limbs preoperatively and at 20 weeks postoperatively. O:C = operated vs. control; O:CL = operated vs. contralateral forelimb; O:IL = operated vs. ipsilateral forelimb. **c** Ratio of impulse for operated limb compared to all other limbs preoperatively and at 20 weeks postoperatively. **d** Ratio of maximum peak pressure for operated limb compared to all other limbs preoperatively and at 20 weeks postoperatively. **e** Change in distribution of bodyweight to each limb at 20 weeks postoperatively compared to preoperatively, and between forelimbs (FL) and hind limbs (HL). *Different from preoperative; *bars* having different *letters* are significantly different

Gait velocity was not significantly different between 0 and 20 weeks postoperatively (2.32 ± 0.08 m/s and 2.32 ± 0.1 m/s, respectively), the two time points for which full datasets were available for analysis. Duty factor did not change in the hind limbs between preoperative and 20-week postoperative values, but increased in the forelimbs, particularly in the contralateral forelimb (diagonally opposite operated hind limb) postoperatively (Fig. 4a). The ratio of peak vertical force (Fig. 4b) and impulse (Fig. 4c) for operated:control hind limbs was significantly decreased at 20 weeks postoperatively compared to preoperatively. As expected, this ratio was also significantly greater between the operated and control hindlimbs than operated:contralateral forelimb or the operated:ipsilateral forelimb. These latter ratios did not change significantly in the postoperative period. The ratio of maximum peak pressure (Fig. 4d) of operated:control was increased compared to other comparisons, but there was no effect of postoperative time. Change in distribution of bodyweight was significantly different at 20 weeks compared to preoperative values (Fig. 4e) for all limbs except the control hind limb. Body weight distribution was significantly reduced to the operated limb and ipsilateral forelimb compared to the control hind limb and the contra-lateral forelimb at 20 weeks postoperatively compared to preoperatively.

### Histological analyses

**Fig. 5:**
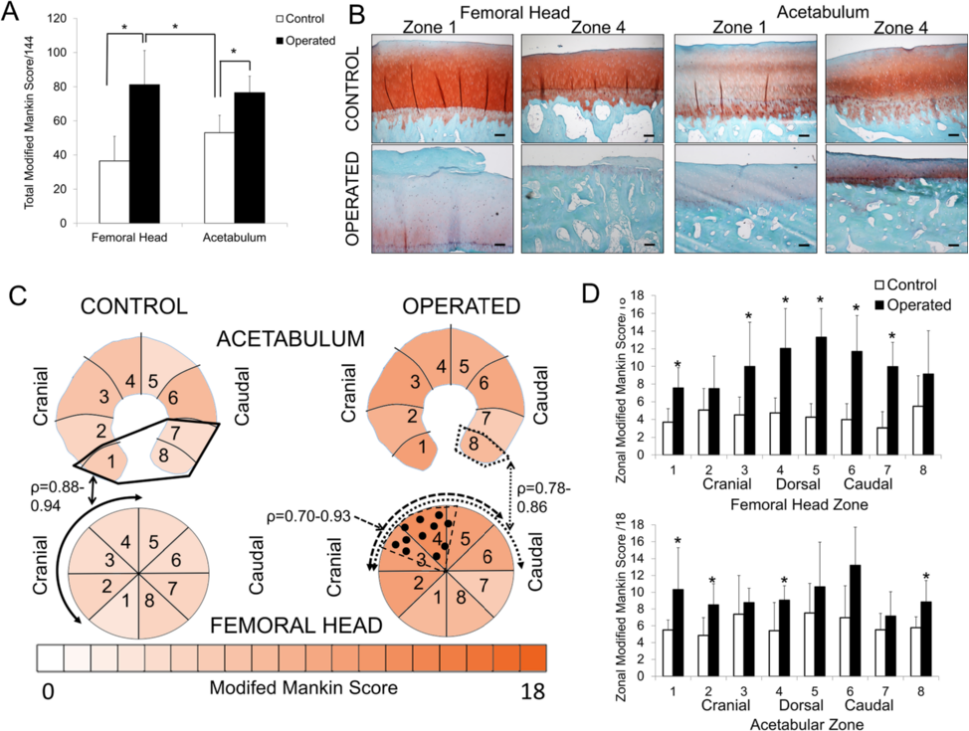
**a** Total modified Mankin scores for femoral head and acetabulum in control and operated hip joints, 20 weeks after surgery. *p = 0.03. **b** Safranin O/Fast Green images of the articular cartilage of zones 1 and 4 of the femoral head and acetabulum in control and operated hip joints. *Scale bar* = 100 μm. **c** Schematic of the mean modified Mankin scores by joint region in control and operated femoral head and acetabulum, with significant correlations between femoral head zone 2–4 modified Mankin score and acetabulum zone 1, 7 & 8 modified Mankin score identified for control limbs (*solid lines* --------), between operated limb femoral head zones 3–6 and corresponding acetabulum zone 8 (*dotted lines* ……), and between zones 3–5 on operated femoral head (*dashed lines* - - - - -). The region of debrided cartilage on operated femoral head is delineated by *dashed lines*/*dots* in zones 3–5. **d** Modified Mankin scores by femoral head and acetabulum regional zone for control and operated limbs. *Operated different from control

**Fig. 6:**
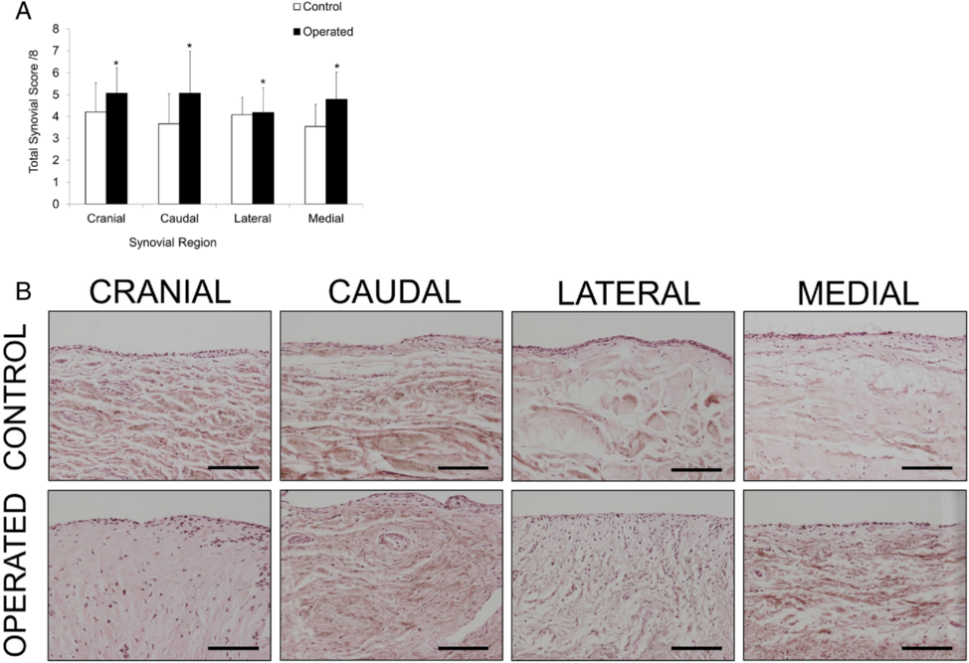
**a** Regional synovitis score for control and operated limbs. *p = 0.05 for control vs. operated. No individual region effect. **b** Representative histological images (H&E) of synovium from cranial, caudal, lateral and medial aspects of the hip joint in control and operated limbs. *Scale bar* = 100 μm

Total modified Mankin scores of the femoral head and acetabular cartilage were significantly greater in operated compared to control limbs, and femoral modified Mankin score was significantly correlated with acetabular modified Mankin score (*ρ* = 0.82, *p* < 0.05) (Fig. 5a and b). Analysis of these degenerative changes by circumferential zone showed that the modified Mankin scores of the operated femoral head were significantly greater than control in the region of surgical debridement of cartilage, and were significantly correlated in zones 3–5 of the femoral head (Fig. 5c and d). Surgical debridement of femoral head cartilage resulted in an increase in modified Mankin scores across fewer zones of the acetabulum compared to the femoral head, and changes in the acetabulum were distributed in general more peripherally (zones 1, 2, 4 and 8) than in the femoral head (zones 1, 3–7). In agreement with this finding, positive correlations between modified Mankin scores of the femoral head and acetabulum were only found for zones 3–6 (operated) and 2–4 (control) of the femoral heads compared to zone 8 in the operated and zones 1,7 and 8 in the control acetabulum (Fig. 5c). Synovial histological scores were significantly increased (*p* = 0.05) in operated compared to control limbs, but there was no effect of region (Fig. 6a and b). Synovial histological scores were correlated with both femoral head (*ρ* = 0.64, *p* < 0.05) and acetabulum (*ρ* = 0.58, *p* < 0.05) modified Mankin scores of all joints evaluated.

### Correlations between outcome measures

Many clinical and radiographic parameters were correlated to other clinical, radiographic and end-point histological parameters. Those of greatest biological interest related to the operated limb are shown in Table 1, and all correlations are shown in Additional file 3: Table S2. Mankin and synovial scores in operated limbs were positively correlated to radiographic scores, but negatively correlated to the range of motion, limb circumference, postoperative body weight, bodyweight distribution to operated limb, maximum force and maximum pressure on operated limb. There were positive correlations between 20-week hip laxity (DI), joint congruency (CI) and overall joint radiographic scores in the operated limb, which were related to radiographic scores at the beginning of the study. None of the accelerometry parameters at 20 weeks correlated with end-point histological or radiographic measures for the operated limb, except for a negative correlation between CI in operated limb at 20 weeks and the number of night time activity bouts at 20 weeks.

**Table 1:** Spearman rank order correlations related to the operated limb at experimental end-point between various biologically relevant clinical and end-point measures (*p* < 0.05), at either 0 or 20 weeks, or the difference in operated limb over time, or between operated and control hind limb

Clinical factor	Clinical or **end**-**point** factor	ρ
Change in bodyweight 0–20 weeks	**Operated total joint & femoral head Mankin score**	−0.82– −0.79
Change in bodyweight 0–20 weeks	Range of motion operated limb at 20 weeks	0.89
Range of motion operated limb at 20 weeks	**Operated total joint and femoral head Mankin score**	−0.96– −0.93
Range of motion operated limb at 20 weeks	Maximum peak pressure, force and impulse O:C at 20 weeks	0.82–0.89
Range of motion operated limb at 20 weeks	Impulse O:C 20 weeks	0.82
Difference in range of motion between operated and control 20 weeks	**Operated acetabulum Mankin score**	−0.93
Difference in range of motion between operated and control 20 weeks	Bodyweight distribution to operated limb at 20 weeks	0.89
Limb circumference operated limb	**Operated mean synovial score**	−0.86
Limb circumference operated limb	**Control mean synovial score**	0.79
CBPI 20 weeks	Bodyweight distribution change hind limbs 0–20 weeks	−0.77
CBPI 20 weeks	Bodyweight distribution change forelimbs 0–20 weeks	0.77
CBPI 20 weeks	Bodyweight distribution to operated limb at 20 weeks	−0.85
Bodyweight distribution to operated limb at 20 weeks	**Operated acetabulum Mankin score**	−0.82
Bodyweight distribution change operated 0–20 weeks	**Operated mean synovial score**	−0.93
Bodyweight distribution to operated limb at 20 weeks	**Control limb acetabulum Mankin score zone 2,6**	0.79–0.90
Bodyweight distribution to operated limb at 20 weeks	Bodyweight distribution to operated limb at 0 weeks	0.86
Bodyweight distribution change operated 0–20 weeks	Impulse O:C at 20 weeks	0.79
Maximum force O:C 20 weeks	**Operated total joint and femoral head Mankin score**	−0.86– −0.79
Maximum force O:C 20 weeks	**Control femoral head Mankin score**	−0.83
Maximum force O:C 20 weeks	Impulse O:C 20 weeks	0.96
Maximum peak pressure O:C 20 weeks	**Operated total joint and femoral head Mankin score**	−0.96– −0.93
Radiographic score operated 20 weeks	**Operated mean synovial and total joint Mankin score**	0.77–0.79
Compression index operated 20 weeks	**Operated mean synovial score**	0.82
Compression index operated 20 weeks	Distraction index operated 20 weeks	0.86
Compression index operated 20 weeks	Radiographic score operated 20 weeks	0.77
Compression index operated 20 weeks	Mean number night activity bouts 20 weeks	−0.79
Distraction index operated 20 weeks	Radiographic score operated 0 weeks	0.94

Notes: *CBPI* canine brief pain inventory, *O:C* operated vs. control

## Discussion

The findings of this study demonstrate that debridement of cartilage from the cranio-dorsal aspect of the femoral head in the canine hip induced moderately severe osteoarthritis and impaired function as assessed by a wide range of measurements, including cartilage and synovial histopathology scores, deterioration in radiographic scores, limb circumference, range of motion, CBPI, accelerometry, and gait analysis. This model also allowed maintenance of joint stability postoperatively, which will be critical for future evaluation of novel therapies. Further, several important correlations between histological and radiographic scores and functional outcome measures were identified. Use of these functional outcome measures in research models of hip osteoarthritis may result in improved predictive validity compared to other models previously used for evaluation of different therapeutic approaches. Changes in several of the functional outcome measures were comparable to those previously identified in dogs with naturally occurring hip osteoarthritis (Lascelles et al, 2010a; Seibert et al. 2012); thus, findings from these and future studies could be compared to a data from a large population of preexisting canine clinical cases. Further, the large number of clinical and biologically relevant correlations between functional, clinical and end-point outcome measures applied to this model suggest that continued use of complex panels of outcome measures in animals models could contribute to improved understanding of how well animal models of osteoarthritis represent the complexities of clinical disease in both human and animal subjects (Belshaw, 2016; Sharkey, 2013).

Animals lost a maximum of 5 % bodyweight at 4 weeks postoperatively but regained weight over the remainder of the study. This finding was mirrored by a decrease in limb circumference in control limbs at 4 weeks postoperatively, suggesting some effect of body weight on limb circumference in control limbs in the immediate postoperative period. Muscle mass was not evaluated in the study, but there was divergence in limb circumference between control and operated limbs that was maintained for the majority of the study, even after the recovery of bodyweight. Progressive decrease in limb circumference would be expected with progressive reduced use of the limb and muscle atrophy as osteoarthritis develops (Anson et al. 1988). We found no correlation between change in bodyweight and change in limb circumference, and dogs were fed to maintain body condition score, an estimate of subcutaneous fat deposits, suggesting that the loss of circumference in operated limbs was not associated with loss of subcutaneous fat deposits but may be a result of distal limb muscle loss. Sarcopenia of lower limb muscles is an area of emerging interest in human hip and knee osteoarthritis, where complex interplay between osteoarthritis, pain, muscle mass and function is the subject of increasing investigation (De Ceuninck et al. 2013). In support of this concept, we found that increasing synovitis scores were correlated to greater reduction in limb circumference. Interestingly, absolute bodyweight at time of sacrifice was not correlated to modified Mankin score of the operated hip, but higher modified Mankin scores in the femoral head were associated with greater postoperative bodyweight loss. Weight loss is recommended for management of osteoarthritis (Nelson et al. 2013) and results in reduced clinical lameness in dogs with hip osteoarthritis (Impellizeri et al. 2000). However, weight loss has also been associated with polyarticular inflammatory joint pain in people (Mies Richie and Francis 2003). This finding suggests that in dogs with normal body condition score that are subjected to an acute intervention to induce osteoarthritis, weight loss may be a sign of pain or functional impairment, and may not necessarily delay development of osteoarthritis or clinical signs of disease.

The immediate reduction in hip range of motion postoperatively suggests an effect of the surgery itself, possibly as a result of the capsulorrhaphy, although this has not been documented previously. Nonetheless, after recovering to preoperative levels by 8 weeks postoperatively, range of motion again deteriorated. At the time of euthanasia, greater reduction in range of motion was correlated with increase in modified Mankin scores of the operated limb, suggesting that goniometry may be a useful outcome measure in monitoring severity of osteoarthritis in this dog model, as has been shown in humans and other animal species (Lascelles et al. 2012; Holla et al. 2011). Evaluation of range of motion in sedated rather than conscious dogs may have influenced results. However, in healthy dogs there is no significant difference between values obtained in sedated compared to non-sedated animals (Jaegger et al. 2002).

The two functional scoring outcomes evaluated in the study fulfilled their intended purpose to some extent; the CMPS-SF was designed using psychometric principles and is a spontaneous and evoked behavioral response scale for assessment of acute pain and need for further analgesic intervention. In this research study, peak mean scores in the first 12 h postoperatively (4 ± 1.83) were similar to the overall values reported in a validation study assessing the intervention level for additional analgesia in a broad spectrum of post-surgical clinical cases (Reid et al. 2007), suggesting that the CMPS-SF may also be useful for assessing acute postoperative pain in research dogs. No additional analgesia was deemed necessary for management of postoperative pain in these animals; therefore, the appropriate intervention level for additional analgesia in research animals could not be determined from these data. The CBPI was designed using psychometric principles to evaluate caregivers’ perceptions of the impact of chronic osteoarthritis on everyday activities and function of their dogs (Brown et al. 2007). In this study, although total CBPI scores (2.67 ± 4.14) at study end-point did correlate to changes in bodyweight distribution between forelimbs and operated limbs, CBPI scores did not correlate with histopathologic scores and were much lower than those previously reported at the beginning of several drug intervention studies for canine osteoarthritis (total CBPI scores 28–64) (Wernham et al. 2011; Brown et al. 2008). The reasons for these differences are not known, but may be associated with incomplete ability to assess the full functional capacity for each part of the CBPI instrument for dogs in research housing, or different perception of normal research animal behavior, pain and function by caregivers compared to these perceptions of companion animals by their owners. Alternatively, the mechanism of pain and interference in function demonstrated by dogs subjected to surgical induction of osteoarthritis may be different to those with naturally occurring osteoarthritis, although this is less likely because median total CBPI scores for dogs with bone cancer are comparable to those with osteoarthritis, and specific disease has little effect on human brief pain inventory scores (Brown et al. 2009; Masselin-Dubois et al. 2013). Therefore, since in this study the values obtained during evaluation of the CBPI did not reflect the values expected from naturally occurring disease, modification of the CBPI may be required for future studies in research animals.

Surgical induction of osteoarthritis induced a biphasic response on daytime activity levels assessed by accelerometry. There was an initial reduction in daytime activity until 4 weeks postoperatively, coincident with a reduction in nighttime activity bouts followed by a second reduction in daytime activity beginning at 16 weeks postoperatively. The immediate decrease in daytime activity could be consistent with mild sedative effects of 3 weeks of postoperative analgesia or of residual postoperative pain, but the second decrease occurred at a time when radiographic osteoarthritis scores were most severe, suggesting that progression of osteoarthritis may have reduced daytime activity levels. In support of this, at 20 weeks postoperatively, there was disruption of the preoperative circadian activity levels during both the light and dark cycles, similar to that reported previously in osteoarthritis intervention studies in dogs and cats (Wernham et al. 2011; Lascelles et al. 2010a). Surprisingly, accelerometry parameters at 20 weeks did not correlate with end-point histological or radiographic parameters. However, dogs with higher radiographic scores preoperatively rested more often during the day preoperatively, but when additional orthopedic disease was superimposed on the existing level of disease by the surgical model, these dogs were more active and rested less during the day at 20 weeks postoperatively (Additional file 3: Table S2). These data suggest that dogs affected primarily with osteoarthritis in the surgical limb became less active after induction of osteoarthritis in that limb, and may fatigue more readily or rest more often, but that those in which the surgically induced osteoarthritis was superimposed on preexisting radiographic evidence of osteoarthritis due to hip dysplasia became more active after surgery, suggesting that they became more restless with bilateral hindlimb osteoarthritis, possibly due to pain. These data may also explain the two distinct behavioral patterns identified when individual circadian patterns and activity levels were evaluated (Additional file 2: Figure S1). Together, these data suggest that surgical induction of osteoarthritis in these dogs may have caused some degree of sleep disturbance, as is seen in humans (Hawker et al. 2010; Allen et al. 2008). The animals in this study were not specifically evaluated for fatigue, spontaneous activity after a fatigue test, restlessness or sleep disturbance, but this is currently under investigation. In humans, fatigue reported by the chronic pain coping inventory has been associated with lower activity levels (Murphy et al. 2012), and has been assessed by activity responses to standardized tasks (Schepens et al. 2012). Sleep disturbance is commonly reported with osteoarthritis, although the pathogenesis may be complex (Leys et al. 2013; Taylor-Gjevre et al. 2011).

Gait analysis has been well validated both in humans and in dogs as an index of joint pain. The increase in duty factor in forelimbs, particularly of the contralateral forelimb, and the decrease in ratio of peak vertical force, impulse and peak pressure of operated:control hind limbs are all consistent with development of osteoarthritis, and the reverse of gait parameter changes identified during treatment of hip osteoarthritis in dogs (Budsberg et al. 1999; Seibert et al. 2012). The percent change in bodyweight distribution between control and operated hind limbs at 20 weeks postoperatively was similar to the difference between preoperative values in dogs with naturally occurring hip osteoarthritis undergoing hip arthroplasty (Seibert et al. 2012). The lack of correlation between radiographic scores and gait parameters may be explained by the relative insensitivity of radiography in detection of changes in the joint influencing functional gait parameters since symptoms of osteoarthritis correlate poorly with radiographic osteoarthritis (Nelson et al. 2014). Magnetic resonance imaging could be evaluated for improved correlation, as has been seen in the canine knee (Moreau et al. 2013), and in this study, histologic parameters did correlate with gait parameters at the study end-point, in agreement with the findings of other studies (Mendes et al. 1974; Skinner and Mabey 1987).

The severity of histological evidence of osteoarthritis correlated between femoral head, acetabulum, and synovium and was most severe in operated limbs in the region of cartilage debridement over the dorso-lateral aspect of the femoral head. However, cartilage debridement did not increase modified Mankin scores consistently in the regions of the acetabular cartilage in direct contact with these lesions. Instead, increases in osteoarthritis scores of the acetabulum were most prominent in the caudoventral region of the acetabulum, highlighting the importance of evaluation of the whole joint (Cook et al. 2010). In contrast, in naturally occurring osteoarthritis in the control limbs of these dogs, there was significant correlation between the cranio-dorsal femoral head and both the cranio-ventral and caudo-ventral acetabulum modified Mankin scores, suggesting that subtle differences in the distribution of osteoarthritis lesions between femoral head and acetabulum between naturally occurring and experimentally induced disease could be detected by this method. Both total joint modified Mankin score and synovitis scores correlated with radiographic scores of osteoarthritis, suggesting that radiography is useful in monitoring structural severity of osteoarthritis in this model. This is similar to naturally occurring disease in other animal models (Jimenez et al. 1997), and in some, but not all, human investigations (Nebelung et al. 2000; Sunk et al. 2013). Preexisting hip dysplasia and osteoarthritis correlated to 20-week radiographic scores in control but not operated limbs, suggesting that the induced lesion was more important in progression of osteoarthritis in this time frame than any preexisting underlying disease process. However, CIs (congruency) of operated limbs were correlated (Additional file 3: Table S2) to radiographic findings at 20 weeks, and Cis (congruency) and DIs (laxity) were correlated in operated limbs at 20 weeks, suggesting complex interplay between joint congruency, joint laxity and development of radiographic osteoarthritis. These data further highlight the importance of maximizing joint stability and congruency postoperatively, to remove any confounding effect of these factors on evaluation of development of osteoarthritis or wear of any construct implanted in future studies. Reconstruction of the posterior joint capsule after THA in humans is critical for prevention of dislocation, assuming well positioned components and lack of impingement between acetabular and femoral components (Tsai et al. 2008; Van Warmerdam et al. 2011). In dogs, the primary indication for THA is osteoarthritis secondary to chronic subluxation caused by hip dysplasia, hence there is typically chronic joint capsule laxity. However, preexisting soft tissue laxity is a known risk factor for dislocation after THA in dogs (Hayes et al. 2011).

The preoperative finding of hip dysplasia in the majority of dogs used in this study suggests that hip dysplasia may be common in hounds purpose-bred for research, and several groups have suggested that canine hip dysplasia is a complex genetic trait with high prevalence in large breeds. (Bartolomé, 2015) If this type of dog is used in the future to study tissue-engineered cartilage constructs and hip dysplasia is identified pre-operatively, wear patterns of any implant may approximate those seen in the population of human patients expected to benefit most from these technologies, but may also complicate analysis and increase variability of results, particularly of functional outcome measures. Spontaneous cartilage lesions in dogs with hip dysplasia are initiated in the dorsal femoral head, adjacent to the insertion of the ligamentum capitis femoris, in a similar location to the perifoveal location of cartilage fibrillation seen in human hip osteoarthritis (Maroudas et al. 1973; Burton-Wurster et al. 1999). The full effect of preexisting hip dysplasia on the results of this study is unknown, since no unaffected control group was available for comparison. However, based on radiographic scores as well as DIs (laxity) and CIs (congruency) of control limbs, we were unable to detect any progression of these abnormalities over the 6-month duration of the study. In addition, DI (laxity), but not CI (congruency), was tightly correlated with radiographic score at both preoperative and 20-week time points, for the control limb (Additional file 3: Table S2). These findings are consistent with previous studies (Gold et al. 2009). Interpretation of accelerometry data may have been confounded by preoperative hip dysplasia. For example, increased radiographic score of the control limb was correlated with increased number of preoperative daytime rest periods. Increased number of daytime rest periods preoperatively was associated with shorter total duration of daytime rest periods at 20 weeks postoperatively and increased overall daytime activity. In summary, hip osteoarthritis in dogs leads to complex changes in gait (Bockstahler et al. 2012), and in this study we found complex interactions of preexisting and induced osteoarthritis on functional outcome measures. Therefore, as has been suggested previously (Cook et al. 2010), dogs should be screened for preexisting hip dysplasia and if identified should not be used in feasibility studies to evaluate new joint therapeutics. Conversely, dysplasia is a major cause of hip osteoarthritis in humans and of abnormal joint mechanics in affected hips (Henak et al. 2014). In addition, dysplasia leads to premature wear and acetabular component fixation failure of both stemmed THA and hip resurfacing arthroplasty (Gross and Liu 2011). Therefore it could be argued that use of dogs with preexisting hip dysplasia could be a good model system to more accurately predict wear and failure modes of new joint repair technologies in patients.

There were several limitations to this study. If data were available before the start of the study regarding the prevalence of hip dysplasia in this breed of research dog, then we would have planned the study to compare results between dogs with and without pre-existing hip dysplasia; this would have answered several outstanding questions as to the impact on functional outcome measures of unilateral vs. bilateral hip disease, and the impact of superimposition of an experimental lesion onto pre-existing naturally occurring osteoarthritis in this research model. Specific evaluation of operated limbs for evidence of sarcopenia relative to the control limb was not evaluated in this study, but would be helpful in future studies given increasing interest in humans relating to the interplay of muscle mass, osteoarthritis and limb function. The CBPI was insensitive in this study when applied to research dogs, therefore modification of the CBPI instrument may be required to allow caregivers to better assess dogs less familiar with human interaction in research housing settings. Finally, the large amount of data from functional, clinically relevant, and end-point measures presented in this study captures many aspects of both naturally occurring (due to hip dysplasia) and induced osteoarthritis over a 6-month time frame. The ability of each of these outcome measures to improve predictive validity during pre-clinical evaluation of new treatments was not tested in this study, but use of these outcome measures and the modifications suggested in future studies using this model could provide critical additional data with respect to efficacy and safety.

## Conclusions

In summary, these data provide characterization of a new model of hip osteoarthritis. The responses of various functional outcome measures to induction of osteoarthritis were presented. Their correlation to both preexisting hip dysplasia and to the surgically induced disease will likely prove valuable in improving the predictive validity of hip models of osteoarthritis in the dog and in evaluating new hip resurfacing technologies.

## Abbreviations

CBPI, canine brief pain inventory; CI, compression index; CMPS-SF, Glasgow composite measure pain scale – short form; DI, distraction index, IM, intramuscularly, IV, intravenously, PSW, pressure sensitive walkway; THA, total hip arthroplasty.

## Additional files

Additional file 1: Table S1. Semi-Quantitative Histological and Synovial Grading Schemes for Hip OA. (DOC 39 kb) Additional file 2: Figure S1.
**A**) Circadian activity for dogs with high or low daytime activity levels preoperatively or at 20 weeks postoperatively. (**B**) Area under the curve for total daily activity for dogs with high or low daytime activity levels preoperatively or at 20 weeks postoperatively compared to the group as a whole. (TIF 372 kb) Additional file 3: Table S2. Complete dataset of Spearman rank order correlations performed for all outcome measures. Correlations highlighted in red are significant at p<0.05.(XLSX 133 kb)
