# Supplementary material for: Functional outcome measures in a surgical model of hip osteoarthritis in dogs
Source: J Exp Orthop. 2016 Aug 15;3:17. doi: 10.1186/s40634-016-0053-5 (PMC4987758; doi:10.1186/s40634-016-0053-5)
Supplement: Additional file 1: Table S1. — Semi-Quantitative Histological and Synovial Grading Schemes for Hip OA. (DOC 39 kb) [file 40634_2016_53_MOESM1_ESM.doc]

**Table S1**

**A. Semi-Quantitative Histological Grading Scheme for Hip OA**

| Parameter | Grade | Description |
| --- | --- | --- |
| Articular Cartilage Structure | 0  1  2  3  4  5  6 | Normal  Undulating articular surface but no fibrillation  Minimal/mild superficial fibrillation ( < 1/10 of the articular cartilage thickness)  Fibrillation/clefts/loss of articular cartilage involving superficial 1/3 of articular cartilage  Fibrillation/clefts/loss of articular cartilage involving superficial 1/3 to 2/3 of articular cartilage  Fibrillation/clefts/loss of articular cartilage involving >2/3 depth of articular cartilage  Fibrillation/clefts/loss of articular cartilage to subchondral bone |
| Safranin-O staining | 0  1  2  3  4  5 | Normal  Mild diffuse loss of staining  Loss of staining in < superficial third of articular cartilage  Loss of staining in < superficial half of articular cartilage  Loss of staining in > half of articular cartilage thickness  Loss of staining in all of articular cartilage thickness |
| Chondrocyte Clones | 0  1  2  3 | None  Sporadic appearance of clones  Large number of clones  No residual chondrocytes |
| Fibrocartilage | 0  1  2  3  4 | None  Thin fibrous tissue/fibrocartilage covers articular cartilage  Fibrous tissue/fibrocartilage covers articular cartilage or partially regrows from bone  Thick fibrous tissue/fibrocartilage covers or replaces all articular cartilage  Denuded bone |

A**. Semi-Quantitative Synovial Grading Scheme for Hip OA**

| Parameter | Grade | Description |
| --- | --- | --- |
| Lining Cell Layers | 0 | 1-2 layers |
|  | 1 | 3-6 layers |
|  | 2 | >6 layers |
| Villous hyperplasia | 0 | No villous hyperplasia |
|  | 1 | Irregular intimal layer/short villi |
|  | 2 | Finger-like hyperplasia |
| Sub-intimal cell infiltration | 0 | No cell clusters |
|  | 1 | 1-2 clusters in field |
|  | 2 | >2 clusters in field |
| Subintimal fibroplasia | 0 | No fibroplasia – loose connective tissue, thin capsule |
|  | 1 | Mix of normal loose connective tissue and areas of fibrosis or generalized mild fibrosis, moderately thickened capsule |
|  | 2 | Generalized severe fibrosis and capsular thickening |
