# Supplementary figures and images for: Functional outcome measures in a surgical model of hip osteoarthritis in dogs
Source: J Exp Orthop. 2016 Aug 15;3:17. doi: 10.1186/s40634-016-0053-5 (PMC4987758; doi:10.1186/s40634-016-0053-5)

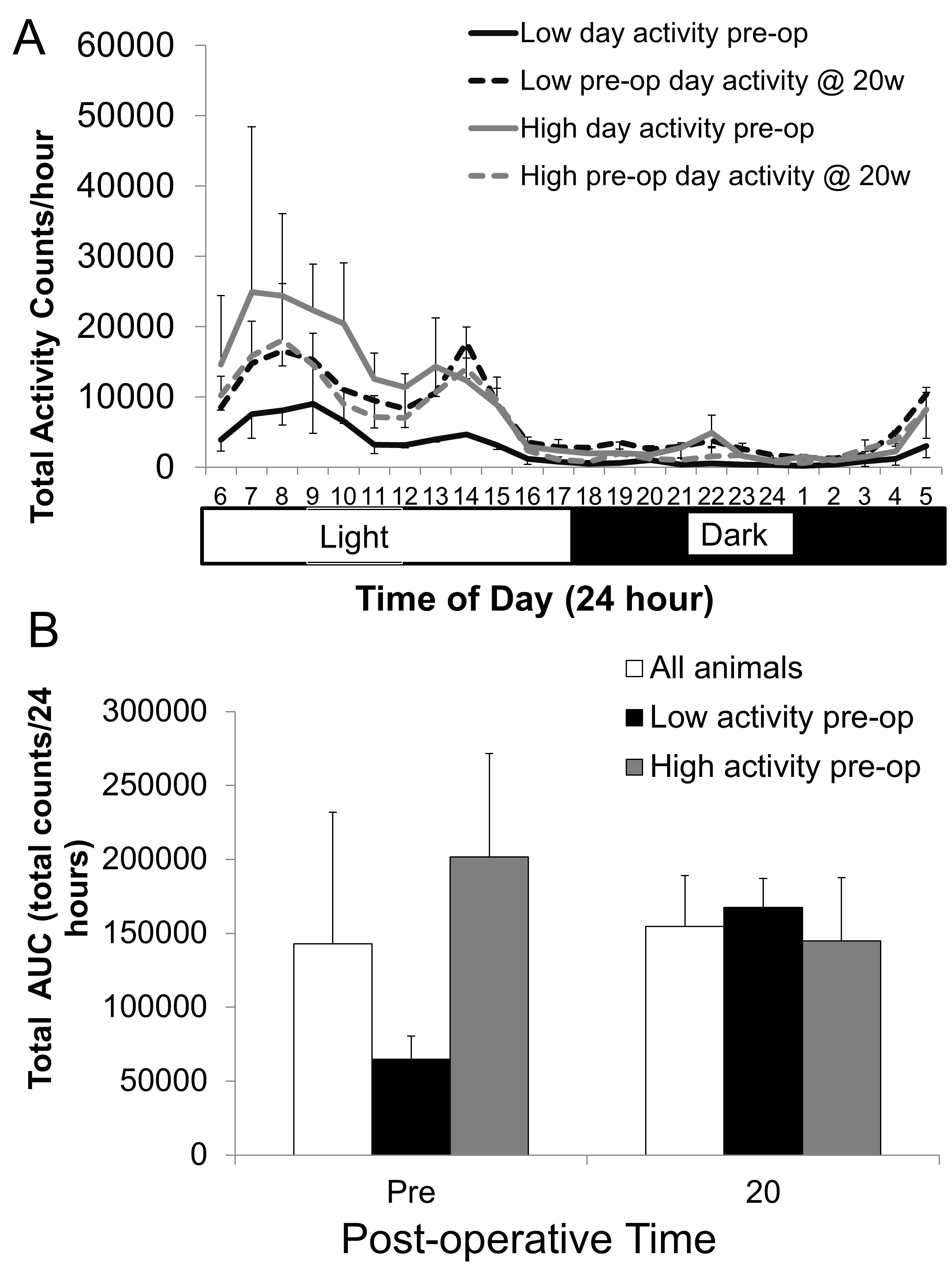

Supplement: Additional file 2: Figure S1. — A) Circadian activity for dogs with high or low daytime activity levels preoperatively or at 20 weeks postoperatively. (B) Area under the curve for total daily activity for dogs with high or low daytime activity levels preoperatively or at 20 weeks postoperatively compared to the group as a whole. (TIF 372 kb) [file 40634_2016_53_MOESM2_ESM.tif]
